# Courtship Initiation Is Stimulated by Acoustic Signals in *Drosophila melanogaster*

**DOI:** 10.1371/journal.pone.0003246

**Published:** 2008-09-19

**Authors:** Aki Ejima, Leslie C. Griffith

**Affiliations:** Department of Biology, National Center for Behavioral Genomics and Volen Center for Complex Systems, Brandeis University, Waltham, Massachusetts, United States of America; Freie Universitaet Berlin, Germany

## Abstract

Finding a mating partner is a critical task for many organisms. It is in the interest of males to employ multiple sensory modalities to search for females. In *Drosophila melanogaster*, vision is thought to be the most important courtship stimulating cue at long distance, while chemosensory cues are used at relatively short distance. In this report, we show that when visual cues are not available, sounds produced by the female allow the male to detect her presence in a large arena. When the target female was artificially immobilized, the male spent a prolonged time searching before starting courtship. This delay in courtship initiation was completely rescued by playing either white noise or recorded fly movement sounds to the male, indicating that the acoustic and/or seismic stimulus produced by movement stimulates courtship initiation, most likely by increasing the general arousal state of the male. Mutant males expressing tetanus toxin (TNT) under the control of *Gr68a-GAL4* had a defect in finding active females and a delay in courtship initiation in a large arena, but not in a small arena. *Gr68a-GAL4* was found to be expressed pleiotropically not only in putative gustatory pheromone receptor neurons but also in mechanosensory neurons, suggesting that *Gr68a*-positive mechanosensory neurons, not gustatory neurons, provide motion detection necessary for courtship initiation. *TNT/Gr68a* males were capable of discriminating the copulation status and age of target females in courtship conditioning, indicating that female discrimination and formation of olfactory courtship memory are independent of the *Gr68a*-expressing neurons that subserve gustation and mechanosensation. This study suggests for the first time that mechanical signals generated by a female fly have a prominent effect on males' courtship in the dark and leads the way to studying how multimodal sensory information and arousal are integrated in behavioral decision making.

## Introduction

Sex pheromones play important roles in the reproductive behaviors of many organisms. These compounds are important for finding a mate, appealing to the mate for successful copulation and also for avoiding inappropriate mates for reviews see [1], [2]. In *Drosophila*, hydrocarbon pheromone profiles also provide more subtle information about a potential mate, e.g. the sexual status of females: their maturation level and/or whether they are previously mated. While both mature virgin and mated females contain aphrodisiac pheromones, mated females have aversive compounds which have been acquired from the male during copulation. Males detect these components and adjust their behavior, showing a reduced level of courtship to copulated females [3]. The hydrocarbon profile of a very young female (∼8 h-old) contains a mixture of saturated and unsaturated hydrocarbons and is very different from that of a mature female (4–5 day-old) [4], [5]. We previously found that a male recognizes the differences between mature and immature females and can produce trainer-type specific courtship suppression upon training with virgin females under conditions in which copulation is prevented [4]. This type of courtship suppression relies on males' formation of an association between volatile maturation-specific compounds and the aversiveness of the failure to copulate, causing a reduction in courtship only toward the type of female used as a trainer.

One of the courtship parameters that is modulated in this learning paradigm is courtship initiation [4]. Courtship was first described by Sturtevant back in 1915 [6], and now is considered to be initiated in response to appropriate olfactory and visual cues emitted by the potential mate, and consists of male orientation, chasing and tapping [7]. Lack of both visual and olfactory information reduces initiation to very low levels [4], [8]. Once courtship is started, gustatory information from the target female contributes, accelerating the courtship ritual and stimulating wing vibrating, licking, curling abdomen and mounting. To date, only one chemosensory receptor, *Gr68a*, has been reported as a putative female pheromone receptor in *Drosophila*. *Gr68a* encodes a gustatory receptor expressed in approximately 10 male-specific bristles of the male's foreleg. Intriguingly, blocking neurotransmitter release by expressing tetanus toxin or RNA interference of the receptor gene under control of a *Gr68a* promoter upstream of a sequence encoding yeast-derived GAL4, lowered both copulation success and wing vibration [9]. These findings suggested that the neurons in which *Gr68a* regulatory sequence is active are involved in information processing of pheromonal cues during late stages of courtship after the male contacts the female. In the current study, we examined whether this group of neurons (which we find includes a wide variety of mechanosensory cells in addition to the previously identified chemosensory cells) also has a role in courtship initiation. The existence of problems at both late and early stages of courtship in *TNT/Gr68a* flies has allowed us to uncover a previously unappreciated role for mechanosensation in initiation of courtship.

## Results

### Courtship behavior of *TNT/Gr68a* mutant males with immobile females is normal

**Figure 1:**
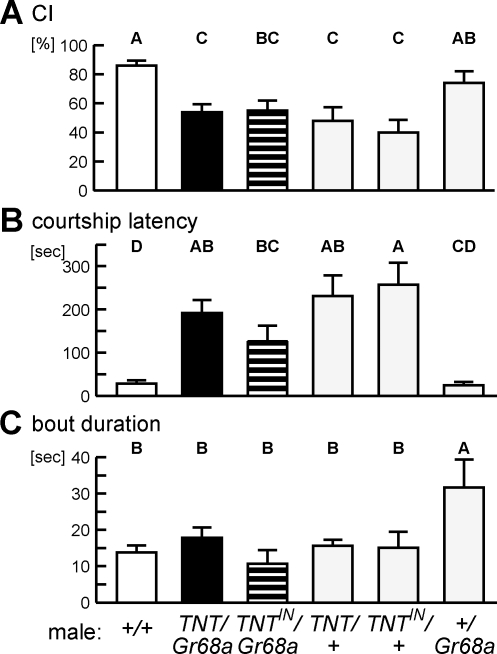
Basal courtship behavior of *TNT/Gr68a* mutant males under standard memory assay conditions. Courtship index (CI, A), courtship latency (B) and bout duration of each courtship (C) were measured for the experimental male (*TNT/Gr68a*, black bar), paired with an immobile, decapitated wild-type female in dim red light. As controls, males expressing an inactive form of TNT (*TNT^IN^/Gr68a*, hatched bar), mono-transgenic heterozygous flies (*TNT/+*, *TNT^IN^/+*, *+/Gr68a*, gray bars) and wild-type (*+/+*, open bar) were also examined. Different capital letters were given above each bar when values were significantly different (*P*<0.05). Behavior was recorded in a round 8 mm diameter ×3 mm depth chamber.

Before examining the role of *Gr68a* neurons in courtship conditioning, we reexamined their role in basic courtship under our standard experimental conditions. In a previous study by Bray and Amrein [9], blocking the output of *Gr68a* neurons caused reduced courtship levels, bout initiation rate and copulation success when intact *w^1118^* females were used as the courtship object in a 300 mm^2^ chamber with room light. For courtship conditioning, in addition to female pheromones, an active courtship performance by the male is thought to be essential during the training session since without a courtship target, exposure to the female extracts alone does not cause modification of subsequent behavior [4], [10]. In some cases, the mobility of the target female can contribute to apparent courtship defects; e.g. courtship level could appear low if the male has visual or locomotion defects that affects his ability to track the moving female, or copulation success could be reduced if the male's courtship is defective and does not stimulate the female to become receptive. To examine the courtship enthusiasm of *Gr68a* males separate from their tracking ability and performance quality, we employed a decapitated immobile wild-type female as a courtship target in a 50 mm^2^ chamber. We also observed behavior under dim red lights, which limits the visual cues available to the male, since visual cues are mostly positive and sometimes dominate over subtle changes and/or defects in other sensory inputs [8], [11]. Males with defective *Gr68a* neuron function were prepared by crossing *Gr68a-GAL4* and *UAS-TNT* to express tetanus toxin (TNT), a protease that blocks vesicle fusion [12]. A *TNT/Gr68a* male was put together with a decapitated mature female and its courtship level (courtship index, CI), courtship latency, and duration of each courtship bout were measured for a 10 min observation period (Fig. 1). As controls, we also examined males expressing an inactive toxin (TNT^IN^), and *+/TNT* and *Gr68a/+* heterozygotes.

As shown in Figure 1A, *TNT/Gr68a* males performed a significantly lower level of courtship (54±5%, black bar) than wild-type males (83±3%, open bar). Courtship latency, the time lag to the first courtship display after pairing with the female, was significantly extended (Fig. 1B, 192.0±29.0 sec for *TNT/Gr68a*, compared with 28.8±7.2 sec for wild-type). In contrast, bout duration, the average length of each courtship bout, was not affected (*P*>0.05), implying that the mutant males have a defect in finding the immobile female in the dark, not a defect in maintaining courtship once it begins. The specificity of this finding for *Gr68a* cell function, however, was not confirmed since all other control strains except the *+/Gr68a* heterozygous control also yielded similarly low CIs (40%∼55%). No significant difference was found between any combination of *TNT/Gr68a*, *TNT^IN^/Gr68a*, *TNT/+* and *TNT^IN^/+*, and between wild-type and *+/Gr68a* males, indicating that the courtship defects of *TNT/Gr68a* males under our experimental conditions resulted primarily from the genetic background of the *UAS-TNT* lines. We conclude that *Gr68a* neurons do not play a critical role in initiating and performing courtship with an immobile female in the absence of visual input.

### Discrimination of maturation stage of trainer females in courtship conditioning is intact in *TNT/Gr68a* males

**Figure 2:**
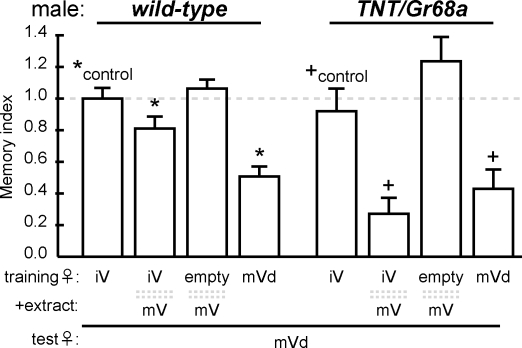
Trainer type-specific courtship conditioning of *TNT/Gr68a* males. Males received 60 min training by exposure to; an immature virgin female (iV), iV and mature virgin (mV) pheromone extract over mesh barrier, mV extract alone or a decapitated mV (mVd), then transferred to a clean chamber and tested with mVd. CI of the tester female was standardized by mean CI of sham to calculate a memory index (see Materials and Methods). All experiments took place in dim red light. Each genotype is compared to own control. * (for wild type) and + (for *TNT/Gr68a*) indicate statistically significant differences (*P*<0.05). Behavior was recorded in a round 8 mm diameter ×3 mm depth chamber.

Males show a high level of initial courtship to a virgin female, but when copulation is prevented over the course of an hour, they lose interest in virgins of that age, and courtship remains reduced up to four hours- this learning has been terms “trainer type-specific courtship suppression” [4]. When a wild-type male is trained with a very young immature female, he will show suppression of courtship toward an immature female [4], but not to a decapitated mature virgin (mVd) tester (Fig. 2 left, *control). In courtship conditioning, virgin female pheromones can be employed as associative cues, so that addition of pheromone extract over a mesh barrier during training changes the specificity, producing courtship suppression with mature female testers too. The extract alone, in the absence of a courtship object produces no suppression (Fig. 2 left). In order to examine whether *Gr68a* neurons are involved in the discrimination of female age, *TNT/Gr68a* males were examined for behavior modification in this paradigm. As shown in Figure 2 right panel, the mutant male produced normal age-specific courtship suppression. Training with a mature virgin produced suppression of mature virgin courtship, but training with an immature female did not change courtship of mature females (+control). Addition of mature female extract over a mesh barrier produced courtship suppression to the mature female. These results indicate that the *TNT/Gr68a* mutant males were able to sense and discriminate the age-specific pheromones of mature and immature females to produce trainer-specific courtship suppression. These cues have been shown to be volatile in nature [4], so these data imply that *TNT/Gr68a* males have normal olfaction, at least for pheromonal cues.

### Suppression of mated female courtship by *TNT/Gr68a* males is normal

It is well established that a mated female elicits less courtship than a virgin female of the same age [3], [13]. To examine whether *Gr68a* neurons are required for the perception of these aversive components of the mated female pheromone, *TNT/Gr68a* mutant males were assayed for their ability to discriminate between virgin and mated females. A mated female that had copulated 24 h before the experiment was decapitated to eliminate active rejection behavior [14], put together with a *TNT/Gr68a* male, and courtship behavior recorded under dim red lights. Compared to a decapitated mature virgin female (mV), the mated female (M) elicited significantly less courtship from the *TNT/Gr68a* mutant (M: 27±6%, mV: 55±9%, *P*<0.05). This suggests that *Gr68a* neurons are not essential to perceive the aversiveness of mated females, and again imply that *TNT/Gr68a* males have normal olfactory processing of pheromones.

### Courtship behavior of *TNT/Gr68a* males toward intact females

**Figure 3:**
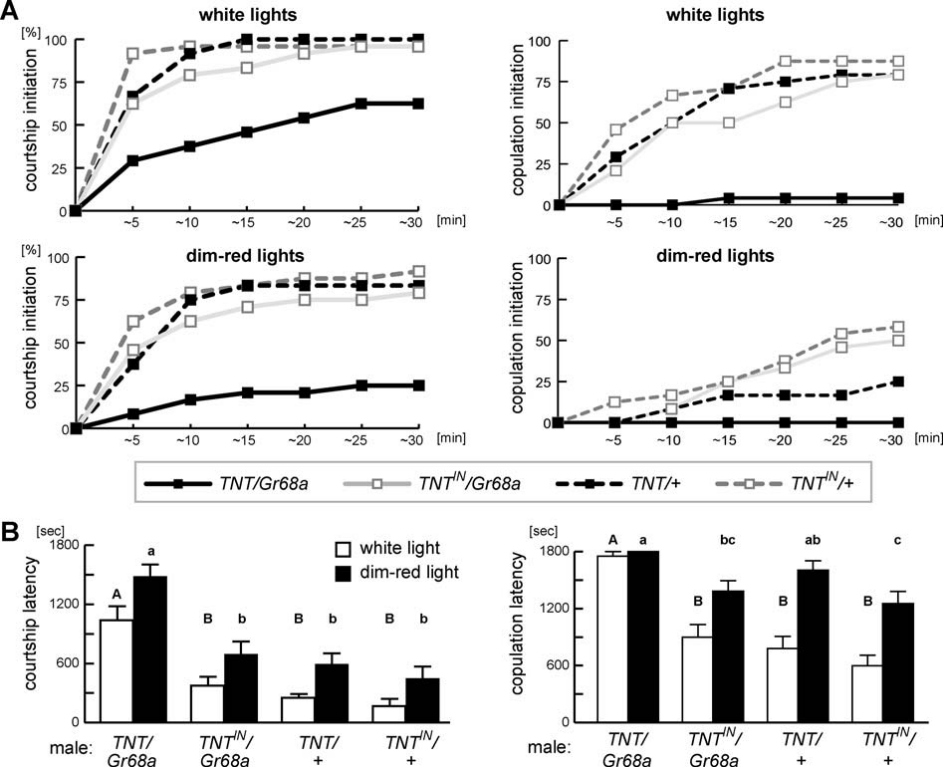
Courtship initiation and copulation success of the *TNT/Gr68a* male in large arena with or without visual cues. (A) Percentage of males that performed the first display of courtship orientation (left) and succeeded mating (right) in either white light (upper panel) or dim red light (lower panel) at each time point during 30 min observation period. (B) Mean values of courtship latency and copulation latency. Statistical significance is represented by capital letters for latencies under white light and small letters for dim-red light above each bar (*P*<0.05). Behavior was recorded in a rectangular chamber with dimensions 70 mm×10 mm×7 mm.

Our finding that *TNT/Gr68a* males showed the same level of courtship as genetic controls (*TNT^IN^/Gr68a*, *TNT/+* and *TNT^IN^/+*, Fig. 1) was unexpected, given the results of Bray and Amrein [9], who showed a role for these cells in both initiation of wing vibration and maintenance of courtship. This suggested that our experimental conditions might mask subnormal male behavior. Our courtship chamber is much smaller (8 mm in diameter, 3 mm in depth) than that of Bray and Amrein (4 mm×10 mm×30 mm). In this limited space, the male could be continuously exposed to positive stimuli from the target female, perhaps overwhelming a subtle change in another modality, e.g. gustation, given the nature of *Gr68a*'s encoded protein. To determine if assay conditions were the basis of differences between our results, we measured latencies of courtship and copulation of individual males in a large environment (7 mm×10 mm×70 mm) with an intact, mobile female. Figure 3A shows a cumulative plot of the percentage of males that initiated courtship at each time point during a 30 min observation. Mean values of courtship latency were calculated for each genotype (Fig. 3B). In room (white) light, only 29% of *TNT/Gr68a* started courtship within 10 min, while over 75% of each of the controls had initiated. No courtship was observed for about a half of the *TNT/Gr68a* males within the 30 min observation period, causing the mean courtship latency to be significantly longer than control (Fig. 3B open bars). Percentage copulation success and the copulation latency for each genotype were also measured; only one *TNT/Gr68a* male (4%) copulated, while at least 75% of each control line was successful, indicating that the *TNT/Gr68a* initiation defect contributed to their inability to copulate. This initiation defect was surprising since it is only after initiation that males obtain gustatory information, and it implies that the *TNT/Gr68a* males have more than a problem with taste.

One possible explanation for the initiation defect of *TNT/Gr68a* males could be that they have a problem in the visual system which prevents them from locating the female. If so, repeating the experiment and eliminating visual input should diminish the advantage of control males and cause them to show the same levels of courtship and copulation as *TNT/Gr68a*. However, the courtship latency of *TNT/Gr68a* males was still significantly longer than any of the controls even in dim red light (Fig. 3). Comparison of white and dim red light latencies implies that males do use visual information for initiation since turning off the lights resulted in significant increases in latency for *TNT/Gr68a*, *TNT/+* and *TNT^IN^/+* (*P*<0.05). None of the *TNT/Gr68a* males copulated during the observation period. This suggests that, while vision is important, the defect of the *TNT/Gr68a* flies did not result from a visual defect.

### *Gr68a-GAL4* is pleiotropically expressed in gustatory and mechanosensory neurons

**Figure 4:**
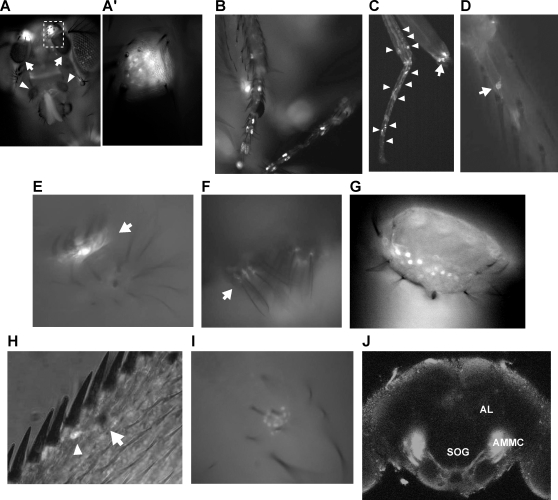
Expression pattern of *Gr68a-GAL4*. (A) External appearance of the GFP expression on a head. Arrows and arrowheads indicate antennal third segment and maxillary palps respectively. (A′) Close-up of the second segment of the antenna. (B) Male forelegs. (C) Female foreleg. (D) Close-up of a male midleg. A cell body (arrowhead) is marked by GFP at base of mechanosensory bristle (arrow). (E) Root of male clasper teeth on the genitalia (arrow) and (F) male anal plate (arrow). (G) Tip of proboscis. (H) Root (arrowhead) of chemosensory bristles (arrow) of the wing margin. (I) Ventral view of female abdomen. (J) Frontal view of a dissected brain, showing antennal lobe (AL), suboesophageal ganglion (SOG), antennal mechanosensory and motor center (AMMC) and optic lobe (OL).

*TNT/Gr68a* males have a significantly extended courtship latency, implying that they have a defect in a modality that is required for finding a female at a distance. Other than vision, the major sensory modality that has been implicated in initiation is olfaction [8]. Although the ability of these flies to form trainer type-specific courtship memory (Fig. 2) under conditions that require olfactory processing suggested that they were normal for this sensory modality, we sought to determine whether *Gr68a-GAL4* is expressed in the olfactory organs, investigating the expression pattern of the driver by using a membrane-bound form of green fluorescent protein mCD8-GFP, [15]. We found no detectable expression on the surfaces of either of the olfactory organs: third antennal segment (Fig. 4A, arrows) or maxillary palp (arrowheads). Interestingly, the second segment of the antenna, which houses Johnston's organ, the fly auditory apparatus [16], showed an intense signal from auditory neurons [17] beneath the cuticular sheath (Fig. 4A′).

*Gr68a-GAL4* was originally reported to have expression in chemosensory neurons of male-specific gustatory bristles on forelegs [9]. This expression was confirmed (Fig. 4B). Female didn't show the male-like foreleg gustatory bristle expression, but they did have multiple GFP-positive cells in the forelegs (Fig. 4C), including three with intensive expression at the base of the leg near where chordotonal organs are located (arrow). This expression pattern was characteristic of all legs of the female and was also seen in the midleg and hindleg of males. A closer look of the male midleg showed cell bodies at the root of mechanosensory bristles (Fig. 4D arrow). The male genitalia contained intense expression at the base of clasper teeth and on the anal plate (Fig. 4E,F). The labellum also exhibited non-sex-specific expression of *Gr68a* at the tip (Fig. 4G) in a region which houses cell bodies of many gustatory neurons [18]. GFP signals were also found at the wing root, the wing margin and on the female abdomen (Fig. 4H,I).

In the brain, expression of *Gr68a-GAL4* is seen in antennal projections that go to a pair of almond-shaped structures known as the antennal mechanosensory and motor center AMMC, [17], which resides next to the suboesophageal ganglion (SOG). Posterior to the SOG, *Gr68a* processes form a commissure connecting the bilateral AMMCs (Fig. 4J). No expression was detected in antennal lobe (AL), which receives olfactory information from the third antennal segment and maxillary palp. In aggregate, we see no evidence of expression of *Gr68a-GAL4* in olfactory structures, but we do find that it is highly expressed in mechanosensory and auditory neurons as well as gustatory structures.

### Female movement stimulates male courtship

The expression of *Gr68a-GAL4* in the second antennal segment and its projection to AMMC in the brain suggests that *TNT/Gr68a* males could have an auditory defect. It is known that courting males listen to their own courtship song (wing vibration) and that this boosts courtship vigor and allows the male to “fine tune” his song [19], [20]. Deaf mutants cannot optimize the frequency of their wing vibration to make high quality courtship song and therefore exhibit low courtship success [21]. While this may contribute to low copulation success of *TNT/Gr68a* males, it cannot explain their increase in courtship latency, since this epoch of courtship occurs before song production. How could an auditory defect affect courtship latency? Does female activity generate specific sounds that attract males? Investigators [22], [23] have recorded a wing-flicking “rejection signal” produced by young females, but did not detect any mature female-generated male-attracting sounds with their recording devices set at 20–2000 Hz, [23]. It is however possible that female movement (foot-steps, grooming or wing flicking) could generate subtle sounds that alert the male to the presence of the female.

**Figure 5:**
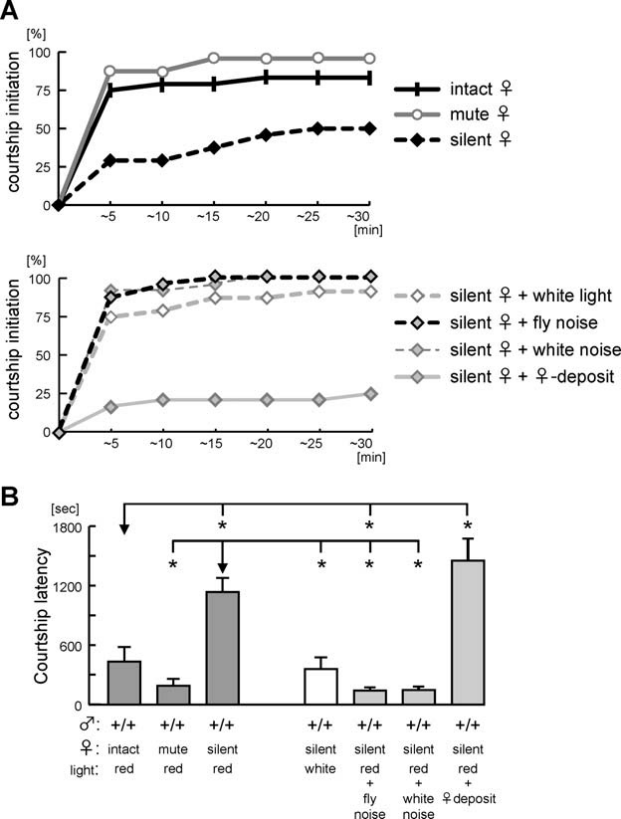
Courtship is stimulated by the active movement of the target female. Wild-type males were paired with the indicated type of female. (A) Courtship initiation was assayed for wild-type, paired with a wingless (“mute”) or a decapitated (“silent”) wild-type female (upper panel) or a “silent” female and additional putative stimulatory cues (lower panel). (B) Mean values of courtship latency in each courtship condition. Mechanosensory stimulus, not excess amounts of female deposits, stimulates courtship initiation. Statistical significance is represented for comparison to the data indicated by each arrow (*P<0.05). Behavior was recorded in a rectangular chamber with dimensions 70 mm×10 mm×7 mm.

In order to test this idea, we prepared two kinds of quiet females: wingless females and decapitated females. The wingless female makes walking and head-grooming sounds, but no wing-grooming, wing-flicking or flight sounds (“mute”). The decapitated female is very still and does not make any active movements except occasional grooming (“silent”). In a large chamber, a wild-type male was put together with either an intact, wingless or decapitated female and was observed for courtship initiation. Figure 5A (top panel) shows that wild-type males had no problem finding wingless females in dim red lights and started courtship as early as with intact females. On the other hand, the lack of active movement caused by decapitation significantly prolonged the courtship latency when there was no visual information (Fig. 5B), suggesting that active movement of both intact and wingless females helped the male to notice the target female. When visual cues are available (white light, Fig. 5A lower panel), males easily find the decapitated silent female, suggesting that the strong positive cue provided by the visual system overcame the lack of female movement.

One possible way in which female activity could enhance initiation is if chemicals released by active females and distributed in the chamber allowed males to use gustatory sensation to “taste” the female's footprints. To test this, we pre-scented the chamber with three active females, but this did not decrease courtship latency toward a decapitated immobile female. It is clear that olfaction is extremely important in initiation in the dark, but these experiments rule out a chemosensory cue as being an explanation for the difference in initiation of courtship toward mobile and immobile targets.

How does female movement stimulate courtship initiation? Does it work as a navigator to help the male to locate the position of the female? Or is it a trigger, which alerts the male to the potential presence of the female, stimulating some sort of searching behavior? In order to assess these possibilities, we provided “fly sounds” to the male in the presence of a decapitated female by placing the courtship chamber over a speaker and replaying a recoding of flies walking around in a chamber. If mechanical stimuli trigger searching, this noise should stimulate courtship initiation. On the other hand, if the test male employs the movement noise of a female to position and/or chase her, noise played through a speaker won't rescue the delayed courtship initiation toward a silent female and might even disrupt the test male's ability to locate the position of the real target female. We found that fly noise enhanced courtship initiation, causing the mean latency of courtship toward a silent female to be significantly shorter than with a silent female alone (Fig 5, *P*<0.0001). The courtship latency toward a decapitated immobile female paired with fly sound was even shorter than that with a mobile female (*P*<0.05). Courtship initiation was enhanced also by addition of a wingless noise maker male into a chamber (*P*<0.05, data not shown), implying that both males and females can emit non-sex-specific mechanosensory signals that alert a male to the presence of another animal in proximity and stimulates him to search for a female in a large field.

The ability of fly sound to stimulate the male to look for a female could reflect either a specific recognition of “fly sounds” or an alteration in the male's attentional state. To discriminate between these possibilities, we played white noise to a male in the presence of a decapitated silent female. White noise was equipotent in stimulating initiation (Fig. 5, *P*>0.8 for comparison to fly noise), indicating that mechanosensory signals are likely to act by increasing the males state of alertness instead of being recognized as specific indicators of the presence or location of another fly.

### Mechanosensory defects in *TNT/Gr68a* mutant males

**Figure 6:**
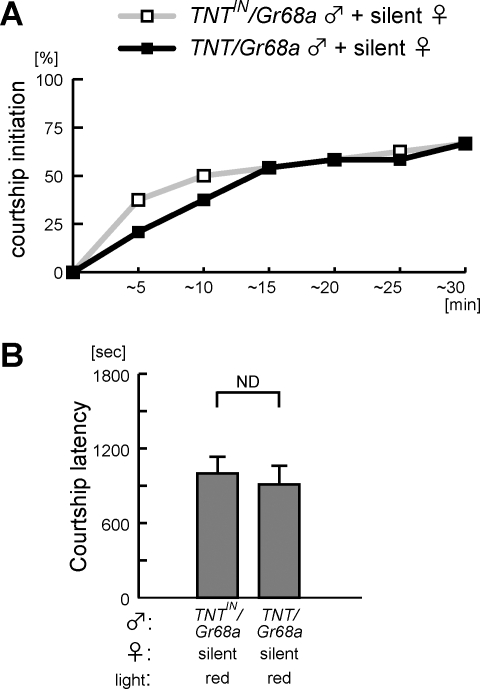
The *TNT/Gr68a* males have no disadvantage in finding decapitated “silent” females. (A) Courtship initiation was examined for the *TNT/Gr68a* mutant males, when paired with the decapitated “silent” females in dim-red light. (B) There was no significant difference between courtship latencies of the *TNT/Gr68a* mutant and the *TNT^IN^/Gr68a* control males (ND). Behavior was recorded in a rectangular chamber with dimensions 70 mm×10 mm×7 mm.

Given the expression of *Gr68a-GAL4* in mechanosensory neurons, it is possible that the *TNT/Gr68a* males cannot detect the female-movement mechanosensory signal, and this therefore delays courtship initiation in the dark. In order to examine this possibility, *TNT/Gr68a* males were paired with a silent female. As shown in Figure 6, there was no significant difference between *TNT/Gr68a* mutant and *TNT^IN^/Gr68a* controls (*P*>0.6), indicating that the *TNT/Gr68a* mutant has no disadvantage when asked to find an immobile female. This suggests that the initiation defect of *TNT/Gr68a* males is specific for mobile females and that TNT expression in *Gr68a-GAL4* auditory and/or other mechanosensory neurons is the cause of poor performance in courtship initiation. This experiment also excludes a possibility that the *TNT/Gr68a* male has a defect in detecting volatile pheromones via *Gr68a*-positive gustatory neurons as a cause of delayed initiation since his performance is equal to that of the control male under conditions in which he must use olfaction to locate the target.

### Function of aristae in courtship initiation

*TNT/Gr68a* mutant males showed a defect in finding active females and a delay in courtship initiation in dark conditions (Fig. 3). *Gr68a-GAL4* is pleiotropically expressed in many parts of fly body (Fig. 4). Which *Gr68a*-positive cells are responsible for the female detection? One of the strongest areas of *Gr68a-GAL4* expression is found in Johnston's organ, the main auditory organ for detection of wing-vibrating courtship song. The song signal is first sensed by an arista attached on the third segment of antenna then transmitted to Johnston's organ inside the second segment [24]. In *Drosophila melanogaster*, *fruitless*, a sex-determination transcription factor, is expressed in most of the auditory neurons of Johnston's organ [25], implying that audition plays an important role in sexual behavior. When a female is exposed to conspecific courtship song, she reduces her locomotion to accept copulation [26]. On the other hand, a male, listening to the courtship song of other males nearby, increases his locomotion and performs enhanced courtship [21], [26].

In order to examine whether males use the arista-Johnston's organ auditory system to detect the moving-female signal, we measured courtship behavior of an auditory mutant, *5D10* [21], and found a defect in courtship initiation in the dark, with a mean latency of 553±185 sec, which was significantly lower than that of its genetic control line, 40A-G13 (control 149±17 sec, *P*<0.005), supporting a role of the auditory system in courtship initiation. We also surgically manipulated arista function and compared courtship responses to those of intact males. Aristae of wild-type males were either fixed to the third antennal segment with a small amount of wax or partially amputated with fine scissors. The males with waxed-arista showed a significantly longer latency of courtship initiation (829±240 sec) than intact males courting intact females (435±193 sec, *P*<0.05). The level corresponded to that of intact males courting decapitated silent females. This was consistent with a freely moving arista being essential for the female-movement detection. However, in this manipulation, the wax also covered some surface area of the third antennal segment so that it is possible that olfactory function was also disrupted, which may cause delayed courtship initiation. Therefore, we next cut most (more than three quarters) of both aristae off with fine scissors. The males with the partial aristae (1/4 arista) showed a normal level of courtship latency compared to intact males (331±100 sec, *P*>0.5). This implies that the full length arista is not essential for motion-signal detection. It is not clear whether the remaining quarter of arista is sufficient for the function or arista itself is not required for this signal detection. It is also possible that Johnston's organ receives mechanosensory signals from other bristles on the second segment and not just the arista. Since *Gr68a-GAL4* is broadly expressed in mechanosensory neurons, it also remains possible that attention-getting auditory stimuli maybe sensed by cells other than those in Johnston's organ.

## Discussion

*Drosophila* males respond to multimodal cues to locate and choose an appropriate mating partner [8], [27]. For dissection of the role of specific sensory modalities, one therefore needs to adjust the experimental conditions e.g. chamber size, illumination and target mobility, in order to preclude strong stimulatory input in other modalities masking subthreshold responses in interest [4], [8], [28]. In *TNT/Gr68a* males, chambers of different sizes led to different courtship performance. In a small chamber designed for learning and memory analysis, *TNT/Gr68a* males generated average levels of courtship (Fig. 1), while in a medium sized chamber, courtship was initiated normally but was maintained only for short period, implying a gustatory defect in the mutants [9]. On the other hand, in a large chamber, mutant males also had difficulty finding females, delaying courtship initiation (Fig. 3). The delayed initiation was a surprising outcome since the *TNT/Gr68a* was reported as a gustatory mutant in the earlier study. Is it possible that a gustatory defect affects courtship performance prior to contact with the female? We investigated the possibility that gustation could be used before initiation (i.e. that the male was tasting the female's pheromone “footprints” in the chamber before he touched her) by pre-scenting a chamber with mobile females before introducing a decapitated silent female, but this did not enhance courtship initiation of wild-type males (Fig. 5). Also, the *TNT/Gr68a* mutant males had no comparable defect in finding “silent” females (Fig. 6). These data led us to suspect that there were non-gustatory defects in *TNT/Gr68a* males and perform comprehensive anatomical characterization of *Gr68a-GAL4* driving a membrane-bound GFP. It was clearly demonstrated that *Gr68a-GAL4* has high expression in auditory and other mechanosensory neurons and in the primary auditory processing area of the brain (Fig. 4). The earlier study missed this pleiotropy, perhaps because a soluble GFP was used as a marker for *Gr68a-GAL4* expression [9]. This supposition, however, leads to the question of whether this GAL4 line is a faithful reporter of *Gr68a* expression, and whether this receptor has a role in mechanosensation. Further analysis using antibodies to the Gr68a protein or a mutant in the gene will be needed. In any case, the robust expression of this GAL4 line stimulated us to uncover a novel role of mechanosensation in courtship initiation.

In this study, we, for the first time, found that acoustic (or perhaps seismic) signals provided by active females stimulate fast localization of the female for courtship initiation. This stimulation of initiation is likely to be due to a change in the attentional state of the male, since noises emitted by either male and female flies or even white noise enhanced initiation. If noise makes the male more alert, he is likely to move around the chamber and to encounter and sense the silent female. *TNT/Gr68a* males failed to detect movement signals and had delayed courtship initiation, suggesting that neural function of *Gr68a*-mechanosensory organs (this broad category includes Johnston's organ, bristles and chordotonal organs) was required for signal detection. However, it is not clear yet whether the signal is transmitted through air as an acoustic signal or as a seismic signal propagating via the chamber floor. The intense expression of *Gr68a-GAL4* in Johnston's organ and AMMC strongly suggests that the auditory system is blocked in the mutant males. Supporting this possibility, an auditory mutant, *5D10* [21] showed delayed courtship initiation in dark. The failure of truncation of the aristae to decrease courtship latency, however, suggests that there may be contributions from other mechanosensory organs to the noise-dependent increase in attention which enhances courtship initiation. Further analysis will be needed to determine whether the mechanosensory system that increases arousal employs an arista-antenna-rotation mechanism [24] as in courtship song detection, or a footstep-sound-transmitted-as-floor-vibration mechanism as do crickets or spiders [29], [30].

## Materials and Methods

### Animal maintenance

Flies were raised on autoclaved cornmeal-yeast-sucrose-agar food, containing the mold inhibitor Tesgosept, in a 12 h light/dark cycle at 25°C. Males and females were anesthetized with CO_2_ on the day of eclosion then used immediately as immature flies or separated by sex and aged for 4 or 5 days. Experimental males were housed in individual tubes. Mated females were prepared by putting 3-day-old females with males. Only females, which copulated for ≥14 min were used the next day. Decapitated flies were prepared by cutting their heads off with fine scissors just before use. For surgical manipulation of the auditory system, aristae of wild-type males were either fixed to the third antennal segment with a small amount of wax or partially amputated with fine scissors (less than one quarter of the arista was left).

### Fly strains

*Canton-S* was used as the wild-type strain. Transgenic lines *Gr68a-GAL4*, *UAS-TNT* and *UAS-TNT^IN^* were provided by H. Amrein at Duke University. *UAS-mCD8GFP* was obtained from Bloomington Stock Center and jumped onto the second chromosome for convenience. An auditory mutant, *5D10* and its genetic control strain, 40A-G13 were kindly provided by D. Eberl at University of Iowa.

### Behavioral assays

All behavior experiments were performed under dim red lights (>700 nm) unless otherwise noted in a Harris environmental room (25°C, 70% humidity). Wet filter paper (Whatman, 42 ashless) was put in each chamber to maintain humidity.

A 4 or 5-day-old male was placed with a decapitated female “courtship object” in a single-pair-mating chamber (8 mm in diameter, 3 mm in depth) and its courtship performance was videotaped with a digital camcorder (Sony, DSR-PD150) for 10 min. A courtship index (CI) was calculated as the proportion of time (×100%) a male displayed courtship action during the 10 min observation period. Courtship latency was the time lag to the first courtship display (courtship orientation) after pairing. A latency value of 600 sec was recorded when no courtship was performed during the 10 min observation. Bout duration is the mean duration of each consecutive courtship sequence between breaks. ≥20 males were tested for each genotype.

For courtship conditioning, a male was put together with a trainer female, either an immature female or a decapitated mature virgin for 60 min. Immediately after training, males were transferred into a clean chamber and paired with a decapitated mature female “tester” for 10 min. Sham trained males were kept alone in the mating chamber for the first hour then paired with a tester. Memory index is calculated by dividing CI at test (CI_test_) by the mean of sham CIs (CI_sham_); CI_test_/mCI_sham_. If memory index = 1, it indicates that there has been no learning since the courtship level of trained males is equivalent to that of sham trained males [13]. ≥20 males were tested for each condition.

For experiments using intact females, a male was paired with a female in a slit-cell chamber (7 mm×10 mm×70 mm) under white lights or dim-red lights and latencies of courtship and copulation, time lags until the first courtship performance or the successful copulation, were recorded. A latency value 1800 sec was given when no courtship or copulation was performed during 30 min observation. 24 males were tested for each genotype and experimental condition.

### Pheromone preparation

When introducing pheromone extracts, a two-part chamber (8 mm in diameter, 6 mm in depth) with a fine nylon mesh (Tetko, 3–180/43) was used to block direct contact with the pheromone-containing filter paper. In order to collect female pheromones, a mature female was put on a wet filter paper in a chamber for 1 h to transfer odors to the filter. For the experiment using female pheromone deposits inside a large chamber (Fig. 5), three intact females were introduced and kept for 10 min before the test then discarded before introducing a test male and a decapitated silent female.

### Sound preparation

Fly sounds (walking and grooming) was recorded from two males and a female in a chamber with courtship song amplifier (Aktogen). Courtship sound (sine song and pulse song) was manually deleted from the recording by observing image references using QuickTime version 7.5 and Amadeus Pro version 1.2.1. The moving-fly sounds and white noise were played back through an audio speaker (Sound Force 660. Intensity was 88∼96 dB at the outside of the 3 mm thick Plexiglas chamber. Background room noise was 78 dB).

### Statistical analysis

Each CI was subjected to arcsine square root transformation to effect an approximation of normal distribution, using JMP software version 5.0.1.2 for the Macintosh. ANOVA with each indicated condition as the main effect was performed on the transformed data. Posthoc analysis was done using Fisher's PLSD test. Bars in figures represent means±SEM with levels of significance indicated by *P significant = 〈<0.05.

### Imaging

Animals expressing *UAS-mCD8-GFP* under control of the *Gr68a-GAL4* driver were observed with a Olympus BX-51W fluorescence microscope. Brains were dissected and imaged on a Leica TCS SP2 mounted on a Leica DMIRE2 inverted microscope without fixation.
